# Insertion and Deletion Processes in Recent Human History

**DOI:** 10.1371/journal.pone.0008650

**Published:** 2010-01-19

**Authors:** Per Sjödin, Thomas Bataillon, Mikkel H. Schierup

**Affiliations:** Bioinformatics Research Center, C. F. Møllers Alle, Århus, Denmark; University of Poitiers, France

## Abstract

**Background:**

Although insertions and deletions (indels) account for a sizable portion of genetic changes within and among species, they have received little attention because they are difficult to type, are alignment dependent and their underlying mutational process is poorly understood. A fundamental question in this respect is whether insertions and deletions are governed by similar or different processes and, if so, what these differences are.

**Methodology/Principal Findings:**

We use published resequencing data from Seattle SNPs and NIEHS human polymorphism databases to construct a genomewide data set of short polymorphic insertions and deletions in the human genome (n = 6228). We contrast these patterns of polymorphism with insertions and deletions fixed in the same regions since the divergence of human and chimpanzee (n = 10546). The macaque genome is used to resolve all indels into insertions and deletions. We find that the ratio of deletions to insertions is greater within humans than between human and chimpanzee. Deletions segregate at lower frequency in humans, providing evidence for deletions being under stronger purifying selection than insertions. The insertion and deletion rates correlate with several genomic features and we find evidence that both insertions and deletions are associated with point mutations. Finally, we find no evidence for a direct effect of the local recombination rate on the insertion and deletion rate.

**Conclusions/Significance:**

Our data strongly suggest that deletions are more deleterious than insertions but that insertions and deletions are otherwise generally governed by the same genomic factors.

## Introduction

Although indels are less common than single nucleotide mutations, they generally account for the majority of differences between species [1]. Indels have also been implicated in many human diseases such as cystic fibrosis, fragile X syndrome and Huntingtons disease [2], [3] as well as in many cancers [4]. Very recently indels have been shown to influence the mutation rate of neighbouring genomic sequences [5]. In spite of this it is our view that, to date, indels have been overlooked in evolutionary studies. This can partly be attributed to difficulties of modelling indels, both because comparatively little is known about their origin and also because a model of indels has to deal with the length of an indel and not only its mutation rate. Indels are also more difficult to handle because they are alignment dependent [6], and indeed, have more often been treated as alignment noise rather than something biologically interesting (but see [7] for an exception).

When considering indels, a fundamental question that needs addressing is whether there is a need to treat insertions and deletions differently. This presents quite a challenge because correctly identifying an indel as either a deletion or an insertion event is very sensitive to alignment errors. It is also obvious that there are major differences in the evolutionary origin and dynamics of indels of small and large size. For instance, large scale indels caused by the proliferation and illegitimate recombination of transposable elements [8], [9] are clearly very different from short indels generated by polymerase slippage, as in micro-satellites [10]. Moreover, some authors suggest that deletions are more deleterious than insertions [11], [12], while others have argued that insertions may be deleterious because they increase the number of sites that can mutate into deleterious variation [13].

Another question to address when looking at indels from an evolutionary perspective is whether there is an association between indels and recombination rate. We believe that there are at least three reasons to expect such an association. The first is a consequence of recombination rates being defined as the number of crossing-over events per physical length unit and since indels by necessity affect the physical length of the sequence region they reside in, they affect this rate. The second reason is more mechanistic: given two homologous sequences the probability of initiating a recombination event in that specific region depends on the similarity of the sequences [14], [15]. In this respect, a length difference between two potentially recombining sequences may be even more likely to inhibit the initiation of a recombination event than a single nucleotide difference. We would, therefore, predict a negative correlation between recombination rates in a small region (*i.e.* one kb) and the heterozygosity of a polymorphic indel in the same region. The third reason is that the recombination process itself could be causing indel formations.

In this study we used polymorphic human data freely available from the University of Washington together with the human-chimpanzee-macaque alignment from the UCSC genome center. We estimate fine scale recombination rates in regions of the human genome covered by our polymorphic dataset. We then examine if the occurrence of polymorphic insertions or deletions affected the recombination in these regions. Also, by using the chimpanzee as outgroup to orient indels polymorphic in the selected human genome sequence, and the macaque genome sequence to orient fixed human and chimpanzee indels, we were able to investigate the dynamics of short (1 to 100 bp) insertions and deletions both on the intra- and the interspecific levels. Contrasting these levels, we gained information concerning differences between insertions and deletions with respect to their origin and genomic effect.

## Results and Discussion

**Figure 1:**
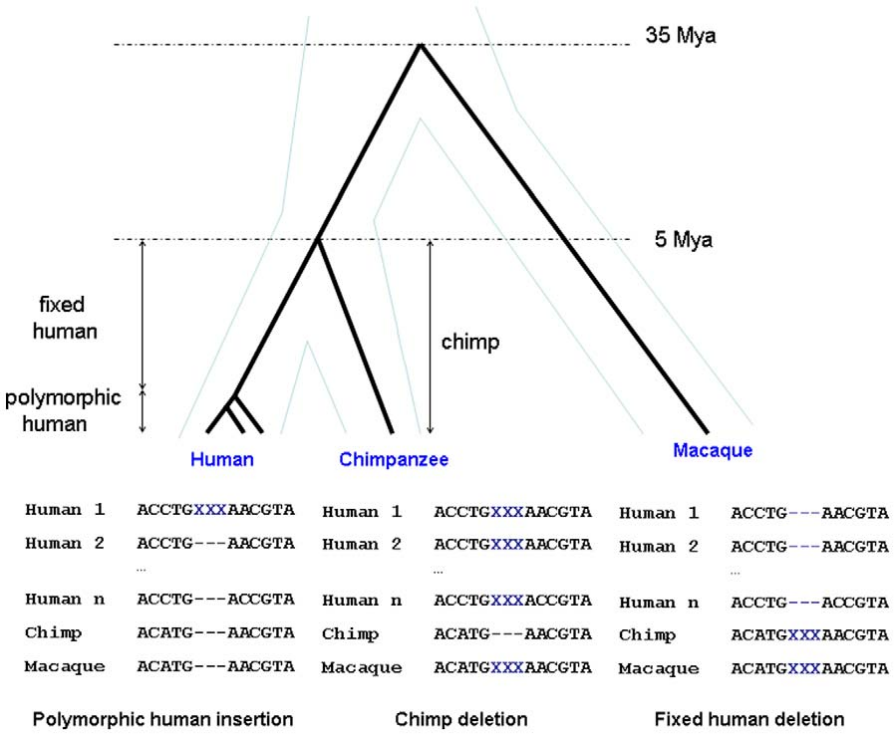
Examples of orientation of indels and definition of data. Dating is approximate and drawing is not to scale. Thin blue lines denote species boundaries and thick black lines denote the gene genealogy underlying the sequence data used in that study.

To compare polymorphic human indels with fixed human indels and chimpanzee indels (set-up shown in Figure 1), we scanned the Seattle SNPs and the NIEHS Environmental Genome Project for indels and SNPs. Then, for the homologous regions scanned for polymorphism, we compared the human, chimpanzee and macaque sequences in the 27 species multiple alignment available from the UCSC genome center to get fixed indel and single nucleotide differences between human and chimpanzee. In total we scanned 20.3 Mb of the human genome (Table 1).

**Table 1:** Summary of data.

	SS	NIEHS	Total
Number of genes	292	599	891
Length	6309228	13995012	20304240
CDS	430037 (6.8%)	987799 (7.1%)	1417836 (7.0%)
UTR	286128 (4.5%)	623147 (4.5%)	909275 (4.5%)
Repeat masked total	3562177 (56.5%)	9791496 (70%)	13353673 (65.8%)
LINE	1451388 (23.0%)	3555778 (25.4%)	5007166 (24.7%)
SINE	1448055 (23.0%)	4478092 (32.0%)	5926147 (29.2%)
LTR-DNA	652622 (10.3%)	1690955 (12.1%)	2343577 (11.5%)
Other	10112 (0.2%)	66671 (0.5%)	76783 (0.4%)

### Sample Frequency Distributions

**Figure 2:**
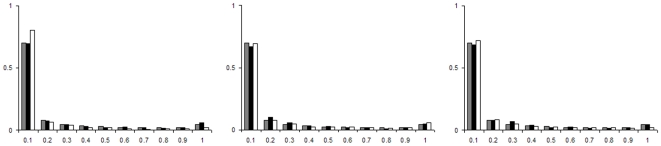
Frequency spectra of indels and SNPs. SNPs in noncoding regions always in grey. These are contrasted to synonymous SNPs (black) and nonsynonymous SNPs (white) in left histogram, to 1 bp insertions (black) and 1 bp deletions (black) in middle histogram and to longer than one bp insertions (black) and deletions (white) in right histogram.

The average sample frequency distributions of SNPs, insertions and deletions are shown in Table 2. In general, deletions have a slightly lower average frequency than insertions. This is also reflected in a significant Wilcoxon rank sum test: polymorphic insertions segregate at significantly higher frequencies than deletions. When comparing, separately, 1 bp insertions to 1 bp deletions and longer than 1 bp insertions to longer than 1 bp deletions, only the latter comparison is significant (Table 3). To get a more comprehensive view of these frequency differences, we also present the frequency spectrum of the derived variant of polymorphic insertions and deletions. We contrast these spectra with those obtained in the same genomic regions for: i) nonsynonymous SNPs, ii) synonymous SNPs and iii) SNPs in noncoding regions (Figure 2).

**Table 2:** Mean sample frequencies.

	Count	Mean
SNPs	70102	0.154
Synonymous SNPs	1851	0.162
Non-synonymous SNPs	1881	0.091
Insertions	1136	0.156
1 bp long ins	697	0.158
Longer than 1 bp ins	439	0.153
Deletions	2714	0.144
1 bp long del	1088	0.161
Longer than 1 bp del	1626	0.133

Mean sample frequency and count of different categories of polymorphic variation in our data. ‘SNPs’ includes SNPs in coding regions. The abbreviations ‘ins’ and ‘del’ are used for insertions and deletions, respectively, in some rows.

**Table 3:** Tests contrasting the distribution of sample frequencies.

Contrast of categories	p-value
SNP nonsyn *vs* SNP syn	<10^−15 ***^
Ins *vs* del	0.0095 ^**^
1 bp ins *vs* 1 bp del	0.19
>1 bp ins *vs* >1 bp del	0.020 ^*^

Summary of tests contrasting the distribution of sample frequencies in various classes of variation (using a Wilcoxon rank sum test).The abbreviations ‘nonsyn’,‘syn’,‘ins’ and ‘del’ are used for nonsynonymous SNPs, synonymous SNPs, insertions and deletions respectively.

### Indel Counts, Ratios and McDonald-Kreitman-Like Tests

**Figure 3:**
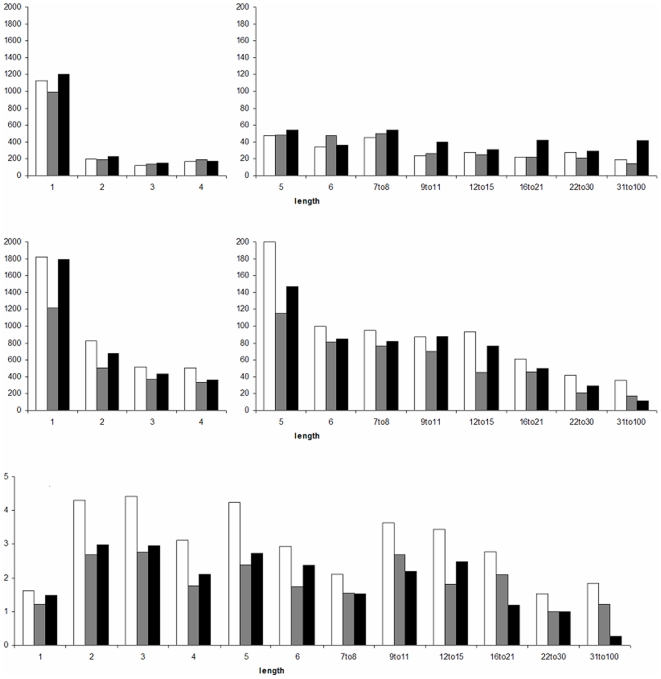
Indel counts. Deletion count (top), insertion count (middle) and DI-ratio (bottom) for different indel lengths (in bp) (white: polymorphic human, grey: fixed human, black: chimp). Note the difference in scale on the y-axis for indels of length 1 to 4 bp and those longer in the two top graphs. Length classes constructed such that the smallest count in any of the six possible indel categories was larger than 20 (except for indels of length 31 to 100 bp for which only the count of polymorphic human deletions and chimp insertions were larger than 20).

The number of insertions and deletions and their ratio are shown in Figure 3 for the three categories and for different lengths.

To test whether the distribution of polymorphic variation is different from fixed human variation we performed a 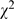
- test on a 2×2 contingency table. This is in essence the same approach as used by McDonald and Kreitman [16] to contrast polymorphism and divergence in synonymous vs. non synonymous sites. We found a ratio of deletions to insertions (*DI-ratio*) of ∼2.4∶1 for polymorphic human indels, similar to that reported by Bhangale et al [17] and the Human Gene Mutation Database. This ratio is significantly higher than for fixed human indels (Figure 3 and Table 4). The ratio of non-synonymous to synonymous changes also mirrors this pattern (Table 4), which suggests that in these ratios, deletion events and non-synonymous changes are the more deleterious variants.

**Table 4:** McDonald-Kreitman tests.

	Nonsyn.	Synonymous	Ratio	Deletions	Insertions	Ratio
Fixed Human	1126	1860	0.61	2893	1753	1.65
Polym Human	2714	2689	1.01	4382	1846	2.37
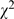	121.49 ^***^			78.67 ^***^		

Adapted McDonald-Kreitman test of differences between non-synonymous and synonymous changes and between insertions and deletions in fixed human and polymorphic human data categories.

The two ratios were also marginally, but significantly, different between the fixed human and chimp categories. This significance disappears (data not shown), however, when the human and chimpanzee sequences from UCSC are compared directly (without dividing the human variation into polymorphic and fixed) as the chimp category also includes some variation that is still polymorphic within the chimpanzee population.

### Genomic Features Affecting the Occurrence of Indels

Results of the linear model analysis of correlates of indels are shown in Table 5. The number of single nucleotide changes in a window strongly correlates with the number of indels and this correlation is exceptionally strong within the “polymorphic” and “fixed” categories. In other words, SNPs are strongly positively correlated with polymorphic indels but not so much to fixed human indels or chimp indels (and so on). The positive correlation between SNPs and polymorphic indels could partly be explained by variation in time to the most recent common ancestor of a window but this effect provides a poor explanation for why the number of indels on the chimp branch seem to be associated with single nucleotide substitutions on the chimp branch while fixed human indels are best explained by single nucleotide substitutions on the human branch. Furthermore, these associations seem to be weaker for AT to GC changes than other single nucleotide changes. This could be a consequence of there being fewer AT to GC changes than other changes, although the difference is only twofold (data not shown). Another strong explanatory factor in common for all indel categories is the presence of poly(A/T).

**Table 5:** Generalized linear model of indels.

	Polymorphic human		Fixed human		Chimp	
	p-value**<**10^−206^		p-value**<**10^−44^		p-value**<**10^−91^	
	Effect	p-value	Effect	p-value	Effect	p-value
Ln(recombination)						
AT→GC SNPs	+	2.5×10^−5 ***^				
Other^a^ SNPs	+	9.8×10^−15 ***^				
AT→GC fixed hum			+	8.2×10^−10 ***^		
Other^a^ fixed hum	+	0.0097 ^**^	+	1.0×10^−10 ***^	+	0.0035 ^**^
AT→GC chimp					+	4.1×10^−4 ***^
Other^a^ chimp			+	0.079 ^.^	+	<10^−15 ***^
GC	−	2.2×10^−5 ***^			−	0.0058 ^**^
polyAT	+	9.6×10^−4 ***^	+	1.3×10^−6 ***^	+	0.015^*^
CpG	+	0.05 ^*^				
%CDS	−	<10^−15 ***^	−	5.9×10^−12 ***^	−	1.8×10^−12 ***^
%UTR			+	0.0083 ^**^	+	0.042 ^*^
SINE	−	1.9×10^−9 ***^	−	0.017 ^*^	−	0.0013 ^**^
LINE						
Repeat masked^b^						
Chromosome X	−	9.1×10^−11 ***^				
Telomere distance	−	0.0074^**^				
Not scanned						
ID	−	2.4×10^−9 ***^			−	1.6×10^−4 ***^
ID : Ln(recombination)	+	0.096 ^.^				
ID : AT→GC SNPs						
ID : Other^a^ SNPs						
ID : AT→GC fixed hum			−	0.064 ^.^		
ID : Other^a^ fixed hum						
ID : AT→GC chimp						
ID : Other^a^ chimp						
ID : GC	+	8.4×10^−4 ***^			+	2.1×10^−4 ***^
ID : polyAT	+	7.8×10^−4 ***^			+	0.0023 ^**^
ID : CpG						
ID : %CDS						
ID : %UTR						
ID : SINE						
ID : LINE						
ID : Repeat masked^b^						
ID : Chromosome X						
ID : Telomere distance			−	0.027 ^*^		
ID : Not scanned						

a single nucleotide changes not AT→GC.

b not due to SINEs or LINEs

Summary of the variables affecting the number of indels in the data. Significance of explanatory variables in generalized linear models for counts of indels are reported. The effect is only reported as decreasing or increasing number of insertions (deletions). An empty box indicates nonsignificance. “ID” indicates whether deletions or insertions are predicted. See text for interpretation of the positive effect of the “ID:GC” and “ID:poly AT” terms.

The number of indels in a window is in general negatively correlated with the percentage of sites in coding exons in a window, but positively correlated with the percentage sites in UTR. Of repetitive elements, only the percentage sites in SINEs is a significant explanatory variable in our linear model. This variable is negatively correlated to all categories of indels.

Although insertions and deletions are generally correlated with the same genomic features, there are some notable exceptions: the highly significant interaction term between indel type and GC-content and poly (A/T) for the polymorphic human and chimp data categories. Here the interpretation of the positive effect of the interaction terms between indel type and GC content and poly(A/T) is not straightforward since this indicates one of two possibilities: either *i*) a stronger positive correlation of GC content (and/or poly(A/T)) with insertions than with deletions, or *ii*) a weaker negative correlation of GC (and/or poly(A/T)) with insertions than with deletions. Closer inspection (by constructing separate models for insertions and for deletions) reveals that *i*) is the cause of the positive effect of “ID: poly AT” while *ii*) is the reason for the positive effect of “ID: GC” (data not shown). This also showed that the marginally significant effect of the interaction between indel type and logged recombination rate is due to a positive correlation between the number of polymorphic insertions and the logged recombination rate in our data.

### Indels and Recombination

The effect of adding *heterozygosity* and the *length effect* of polymorphic insertions and deletions on a trained linear model (see Table S1 for specifications of the trained model) of logged recombination rate are shown in Table 6. A model using only windows with insertions does not show a significant improvement over the trained model but the recombination rate is significantly better predicted in the model fitted only on windows with polymorphic deletions. This could be a consequence of there being many more windows with deletions than windows with insertions. However, a model using all windows with indels (instead of treating insertions and deletions separately), was not significant (data not shown). In the deletion model, we find a negative correlation of length effect and recombination, as predicted. A positive effect of heterozygosity was also found although it was not predicted under our hypothesis.

**Table 6:** Linear model of the log-transformed recombination rate.

	Insertions		Deletions	
	Estimate	p-value	Estimate	p-value
Length *x* (1-long freq^a^)	0.0039	0.65	−0.056	0.0019 ^**^
Indel heterozygosity	0.37	0.51	1.1	0.0065 ^**^
Adjusted R-squared	−0.00085		0.0028	
p-value	0.74		0.0016 ^**^	

a derived frequency if insertion but frequency of ancestral variant if deletion.

Only windows containing polymorphic insertions (1642 windows) or deletions (3837 windows) respectively were used.

### Deletions Are More Deleterious Than Insertions

In many ways the difference between insertion and deletion counts mirrors the difference between synonymous and non-synonymous single nucleotide mutation counts (Table 4). The DI-ratio is much higher for polymorphic human indels than fixed human indels in our data (Figure 3 and Table 4). A sizable proportion of all polymorphisms in the human genome (SNPs, indels and CNVs) are expected to be weakly deleterious and not destined to become fixed. Accordingly, the ratio of non-synonymous to synonymous SNPs is expected to be much higher than the ratio for non-synonymous to synonymous fixed single nucleotide sites. Similarly, the DI-ratio for polymorphic human indels is much larger than for fixed human indels and can, therefore, be interpreted as stronger selection against deletions than insertions.

**Figure 4:**
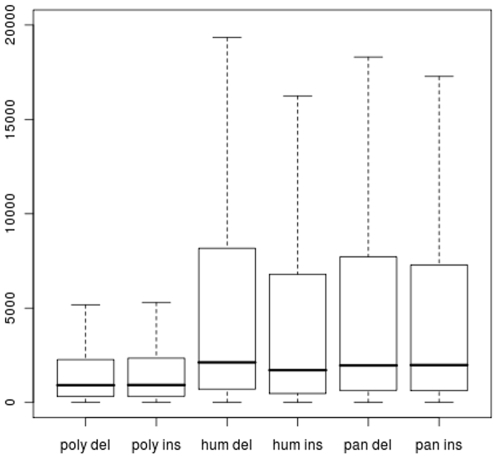
Distance to closest coding region. Boxplots of distance to the closest coding region of polymorphic deletions (“poly del”, n = 4350), polymorphic insertions (“poly ins”, n = 1831), fixed human deletions (“hum del”, n = 5824) , fixed human insertions (“hum ins”, n = 4789) , chimp deletions (“pan del”, n = 7527) and chimp insertions (“pan ins”, n = 3929). Indels within coding regions were excluded.

Additional evidence for deletions being more deleterious than insertions is provided by their sample frequency distributions: polymorphic deletions segregate at significantly lower frequencies than polymorphic insertions (Table 2 and 3). Stronger selection against deletions than insertions has also been suggested in several earlier studies [11], [12], [18], [19]. One explanation is based on deletions requiring two cut points, while an insertion only has one [11]. Briefly, if an important motif resides in sequence positions *n_1_* to *n_2_*, an insertion of any length at position *n_1_* to *n_2_−1* will disrupt this motif. Deletions at these positions will also disrupt the motif but additionally, so will deletions of length ≥*k* at start position *n_1_−k*. This explanation to why deletions may be more deleterious than insertions predicts, first of all, that the detrimental effect of deletions is more length dependent than the detrimental effect of insertions. Our findings that the mean frequency of 1 bp insertions and 1 bp deletions are not significantly different while, despite considerably fewer data points, the mean frequency of deletions longer than 1 bp is significantly smaller than insertions longer than 1 bp (Table 3) thus provide support for this explanation. Moreover (this point is due to an insightful reviewer), assuming that the density of important motifs decreases with distance to coding regions, the difference in selection between the two indels types should be larger close to coding regions than far away. In our data this is reflected by a larger mean and variance of distance to the closest coding region (Figure 4), and also in a stronger negative correlation between sample frequency and distance to coding regions, for deletions than for insertions (Table 7).

**Table 7:** Correlation between sample frequency and distance to closest coding region.

	samplesize	correlation	p-value (Pearson)
Polymorhic deletions	2608	−0.04681	0.0168^*^
Polymorhic insertions	1096	−0.0279	0.3561
SNPs	64207	−0.01418	0.0003^***^

Only variation outside coding regions and with no more than 5% of the sample missing were used.

### Differences between the Origin of Insertions and the Origin of Deletions

If deletions are more deleterious than insertions this suggests that the DI-ratio also correlates with functional constraint across the genome. If this is true, the DI-ratio may provide an alternative method to locate functionally important intergenic regions. However, for this to work it has to be assumed that insertions occur at the same rate as deletions. In our study we found very little that differentiated insertions from deletions except that poly(A/T) and GC content were significantly more correlated with insertions than deletions. Both a high poly(A/T) and a high GC content result in an increased ‘repetitiveness’ and thus the propensity of polymerase slippage. One explanation of this difference is that insertions are relatively more likely to be the outcome of an indel event caused by polymerase slippage than other indel causing events. This is very much related to the dynamics of microsatellites as microsatellites by definition are low complexity regions. We do not wish to specifically discuss microsatellites here (see for instance [10] for a review). Suffice it to say that our data is consistent with the numerous reports that microsatellites tend to expand ( = insert repeats) when short but contract ( = delete repeats) when long and that although our data certainly contains some microsatellites, our filtering procedure should have excluded the majority of long (hyper mutable) microsatellites (see attached material “Data filtering and possible biases” for more details).

While some studies find no association between recombination and indels [5], [20], other authors have reported that insertions are associated with factors linked to recombination while deletions are mostly associated with replication-related features [21]. Our analysis provides little evidence to suggest that recombination has a differential role with respect to insertions and deletions. Although we find a marginally significant positive effect of recombination rate with polymorphic insertions, other expected patterns, assuming recombination promotes insertions but not deletions, such as a significant interaction term between indel type and number of AT to GC mutations (AT to GC mutations should be overrepresented in regions with high recombination rate [22]), are absent (Table 5). That recombination may be positively, but equally, associated with insertion and deletion formation is, however, indirectly supported by our linear model analysis. For instance, the significant positive correlation between UTR content and (both kinds of) indels is possibly a result of an increased recombination rate. Since recombination is known to be suppressed in coding regions, but elevated in flanking regions [23] this could be interpreted as indirect evidence for recombination and indels being positively correlated. This being said, the correlation is rather weak and only significant for fixed human and chimp indels. Moreover, our analysis suggests that any direct causal link between short indels and recombination can be neglected. Where an underestimate of recombination rate in windows with polymorphic indels is by construction a necessity, the effect of this is very weak (Table 6). This is likely due to a lack of statistical power as the variance of the recombination process is known to be very large [24], whereas the underestimate caused by the short indels we study is very small. In fact, given that the distance between SNPs with a polymorphic indel between them is on average shorter than the distance given in the databases (an indel in the Seattle SNPs and NIEHS databases is always represented by the long indel variant regardless of whether it is an insertion or a deletion), we underestimate the recombination rate. Given the average sizes of insertions and deletions in our data, we estimate roughly that the effect of this on our linear model of recombination rate should be smaller than 0.5% of the variance (see Text S1).

Based on a subset of the data analyzed here, Bhangale and coauthors [17] suggested that gene-conversion events between two regions may, compared to SNP differences, be particularly suppressed when there are indel differences. Their suggestion was an attempt to explain their interesting finding that while SNP diversity is significantly greater, indel diversity is significantly lower in repeat masked regions compared to other regions. Although a negative association between repetitive regions (more specifically SINEs) and indels exists in our data, the suggestion that recombination is greatly inhibited by short length differences between sequences seems to be more or less ruled out by our results (Table 6).

### The Occurrence of Indels Is Associated with Single Nucleotide Changes

Our linear model of indels suggests that human SNPs are more closely associated with polymorphic human indels than with fixed human indels for which the strongest association is instead with fixed human single nucleotide differences. Likewise, chimp indels are more strongly correlated with single nucleotide changes on the chimp branch than with any such events on the human branch. In other words, indel events and single nucleotide changes are positively correlated. We interpret these correlations within data categories in three ways: either this is a demonstration of the recent finding that indels are mutagenic [5], or there are mutational hotspots along the genome affecting both single nucleotide changes and indel formation (these would then, similar to recombination hotspots [25], be transient over time), or, as suggested by a reviewer, it is an effect of a local variation in effective population size due to background selection or selective sweeps.

### Underrepresentation of Indels on the X-Chromosome

Kvikstad and coauthors [21] noted that indels are strongly underrepresented on the X-chromosome, which they suggested could be evidence for indel formation by replication errors: the X-chromosome spends less time in a male background, and is thus less affected by cell divisions, than are autosomes. This interpretation is not corroborated by our results as only polymorphic indels are strongly underrepresented on the X-chromosome. Instead we propose that since the amount of polymorphism expected is *4N_e_μ*, where *N_e_* is the effective population size and *μ* is the mutation rate, the lower number of indels could be a consequence of the lower effective population size of the X-chromosome compared to autosomes. There are however several important differences between their study and ours that should be pointed out: their choice of window size is more than 2500 times larger than ours; they do not separate human indels into polymorphic and fixed; they do not consider indels in the chimpanzee lineage; they only study indels in interspersed ancestral repeats and their recombination rates are estimated on a much larger scale than ours (data from Kong et al's map [26]). It should also be mentioned that their data set is an order of magnitude larger than ours.

### Conclusions

What we did find from using human polymorphism and human chimp divergence data was strong evidence for deletions being more deleterious than insertions, and contrary to earlier studies, we suggest that recombination does not play a differential role with respect to insertions and deletions. More generally, and although there definitely exist differences between insertions and deletions [20], [27], our analysis is more in line with earlier findings that these differences may be on a very local scale and can be ignored [28].

Over all, from the data analyzed during this study we believe that by studying the distribution of lengths between indels [7] and by comparing DI-ratios across genomic regions, indels provide unique information for quantifying the amount of functionally important material in different classes of non-coding regions.

## Materials and Methods

### Data Sets

Sequence data for this study was obtained from Seattle SNPs (http://pga.mbt.washington.edu/) and the NIEHS Environmental Genome Project (http://egp.gs.washington.edu/), both made available by the University of Washington. The Seattle SNPs data consists of a 9.9 Mb sequence divided among 301 candidate genes associated with inflammatory response, while the NIEHS dataset consists of 27.5 Mb in 616 genes thought to be involved in environmental response. For genes in both datasets, some parts were not resequenced; these are typically repeat masked regions. In total 21.0 Mb (56.3%) out of 37.4 Mb, were effectively resequenced. We also used the 27 species multiple alignment available from the UCSC genome center (http://genome.ucsc.edu/) to orient variation. After removing genes for which mapping of positions in the data from the University of Washington to positions in the UCSC alignment was problematic, we were left with 891 genes and 20.3 Mb of resequenced data (Table 1).

### Derived and Ancestral States

Using the resequenced data from the University of Washington as a starting point, we retrieved the homologous human-chimpanzee-macaque alignment from the UCSC genome center. We categorized any variation within the data from the University of Washington as polymorphic. Differences between the human and chimp genomes in the human-chimpanzee-macaque alignment that 1) overlapped with the resequenced regions and 2) did not overlap with polymorphic variation were designated as fixed differences. The human-chimp alignment was used to orient human polymorphisms into ancestral and derived states by assuming that the chimp variant was ancestral. In the same way, fixed differences between human and chimp were oriented using the human-chimp-macaque alignment. Orientation is straightforward for segregating sites but slightly more complex for indels. A fixed indel difference can be classified into one of four categories; 1) an insertion on the human branch, 2) a deletion on the human branch, 3) an insertion on the chimpanzee branch, and 4) a deletion on the chimpanzee branch. For instance, if the chimp sequence is the long variant this implies that either a human deletion or a chimp insertion occurred and we need the macaque sequence to resolve this (see Figure 1).

By way of this orientation procedure, we categorized variation as: i) *polymorphic human* data; that is variation found within the Seattle SNPs or NIEHS databases, ii) *fixed human* data; events in the human lineage since the human-chimpanzee split not polymorphic in the Seattle SNPs or NIEHS databases, and iii) *chimp* data; events occurring in the chimpanzee lineage since the human-chimpanzee split (Figure 1). We use this denotation of the categories throughout.

### Frequency of Derived Polymorphic Variation

For each SNP and indel polymorphic within the Seattle SNPs and NIEHS databases the genotypes of the individuals in the samples are provided. Restricting the data to variation with only two variants where one of them is the same as the variant found in the outgroup (see above), the frequency of the derived variant was calculated by dividing the count of the derived variant by the total number of identified variants. In obtaining the frequency spectrum a potential issue is the fact that samples from distinct population are pooled within the Seattle SNPs and NIEHS databases. Moreover, not all polymorphisms were systematically amplified for all individuals in a sample. We investigated the influence of this by binning frequencies in different ways but saw no qualitative differences and we settled for 10 frequency intervals.

### Filtering

First, we corrected some obvious minor errors in the NIEHS and Seattle SNPs data. Alignment artifacts may be responsible for complex patterns of fixed segregating sites and indels overlapping SNPs and polymorphic indels. We conservatively removed such problematic regions in the following way: For SNPs, we required that at least 12 out of the 16 directly adjacent positions should be identical in the human chimp alignment. As the state of indels is much more sensitive to alignment problems, we used a more stringent qualification procedure for these. For polymorphic indels, we required that 28 out of 32 flanking positions were identical in the human-chimpanzee alignment and also that there were no gaps at these positions. We also required that either all human sites were gaps or that all chimp sites were gaps within the indel sites in the human-chimpanzee alignment. For fixed indels, we added the additional requirements that 20 out of 32 directly adjacent flanking positions were identical in the human-chimpanzee-macaque alignment and that there were no gaps at these positions. Again, no ambiguous information was allowed within the indel sites in the human-chimpanzee-macaque alignment. To avoid stronger selection against deletions than against insertions, the only states we considered within the indel sites were ‘gap’ or ‘not gap’ - we did not take into account whether nucleotides within the indel sites matched each other between species. While the complete data cleaning procedure leads to loss of data we believe that data are discarded in an unbiased way with respect to SNP-type and indel-type (see Text S2).

### Estimating Recombination Rate

We used the SNPs provided in the ‘.prettybase.txt’ files provided in the bulk download of both databases with at most 10% missing data as input to the package LDhat [24] to estimate the recombination rate along the genes. Two different programs to estimate recombination rate are available in LDhat: *inter* [29] and *rho* [30]. The program *rho* fits a recombination hotspot process on top of a varying background recombination rate while the recombination hotspot process is not implemented in *inter* [30]. We ran *inter* with the recommended penalty of 5 and *rho* with default parameter values. Since there were no qualitative differences between the estimates from the two programs we report only results using *inter*.

### Statistical Models

The LDhat program used an average recombination rate resolution of 386 bp. We therefore divided sequences into windows of length 386 bp, and calculated the recombination rate in each window as follows. Given that a specific standardized window is overlapped by *n* LDhat-windows with (overlapping) lengths *L_1_*, *L_2_*,…, *L_n_*, and estimated recombination rates *R_1_*, *R_2_*,…, *R_n_*, the average recombination rate of the standardized window was calculated as 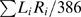 for *i* = 1,‥,*n*. Finally we discarded windows where the percentage of resequenced sites was lower than 90%.

#### Linear models of indels

We constructed generalized linear models with the counts of polymorphic human, fixed human and chimp indels in each window as response variables (three models in total). The explanatory variables we used were the log transformed recombination rate, the GC content, CpG count, the number of bases that are part of a poly (A/T) tract (defined as a contiguous stretch of at least 4 bp with only A's or only T's), the percentage sites in coding regions, the percentage of sites in UTR (in Genbank the designated mRNA which is not coding) and the percentage of sites not scanned. The percentage of sites in repeat masked regions was divided into three categories depending on whether the region was masked due to the presence of SINEs, LINEs or something else. A chromosome X indicator variable and the physical distance of each window to the telomere/chromosome end were additionally used as explanatory variables. Finally, we included the number of single nucleotide differences in each window. These were divided into polymorphic human, fixed human and chimp and also according to whether they were a change of type AT→GC or something else. Instead of having separate models of insertions and deletions, we included an indel type indicator (*ID*, for *i*nsertion or *d*eletion) as an explanatory variable to indicate whether the number of deletions or the number of insertions were to be predicted by the linear model. Each window was used twice: once to predict the number of insertions and once to predict the number of deletions. All interaction terms with this indicator and the explanatory variables above were included. In this way, if one of these variables affected insertions and deletions significantly differently, this should result in a significant interaction term between this variable and the indicator variable. As a consequence of the way we constructed this indicator variable, a positive coefficient of an interaction term between the indel indicator variable and another explanatory variable, *X*, means that *X* should have a larger coefficient to predict insertions than deletions while a negative value of the coefficient of the interaction term implies the opposite, that the coefficient of *X* should be larger for deletions than for insertions. Using a 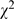
- test, each of the three GLM models were finally compared to a corresponding reduced model without the indicator variable.

#### Linear models of recombination

When investigating how indels relate to the log-transformed recombination rate, all explanatory variables used in the prediction of the indel counts described above were used except for the recombination rate, variables related to fixed human variation and chimp variation. Polymorphic human indels were also treated separately. We trained a model using all windows without polymorphic indels (42214 windows). To do so, all two-way interactions were initially included and the model was subsequently reduced using the stepwise procedure implemented in *R* [31]. To investigate separately different aspects of polymorphic indels, this model was applied to 1): a data set consisting of all windows containing insertions (1642 windows), and 2): a data set consisting of all windows containing deletions (3837 windows). In both cases, the response variable was the log transformed recombination rate in a window minus the predicted value of the trained model for this window.

Since the average distance between two sequence positions is shorter in an alignment including a polymorphic indel, we expected a negative *length effect* of indels on recombination. In order to test for this, we used (1−*p*)*l*, where *p* is the frequency of the long variant and *l* is the length of the indel as explanatory variable (see Text S1). If a length difference between two sequences influences the probability of a recombination event, such an effect would correlate with how often the length variants occur together in an individual. Hence, *p*(1−*p*) was used as a variable to search for this type of effect which we label by *heterozygosity* (see Text S1). When there was more than one deletion, or more than one insertion per window, the maximum of the length effect and heterozygosity value was used. Finally, for the data set with deletions, the length effect and heterozygosity was calculated only from deletions and, likewise, deletions were ignored for the insertion data. All statistical computations described below were performed using the statistical computing language *R* [31].

## Supporting Information

Text S1 Indel recombination model. Derivation of the parameters ‘indel heterozygosity’ and ‘length effect’ used in our linear model of recombination.
(0.09 MB DOC)

Text S2 Data filtering and possible biases. Discussion of possible non-biological sources of our results.
(0.27 MB DOC)

Table S1 Trained recombination model. Table showing specifications of the trained recombination model.
(0.08 MB DOC)
