# Supplementary material for: Insertion and Deletion Processes in Recent Human History
Source: PLoS One. 2010 Jan 19;5(1):e8650. doi: 10.1371/journal.pone.0008650 (PMC2808225; doi:10.1371/journal.pone.0008650)
Supplement: Text S1 — Indel recombination model. Derivation of the parameters ‘indel heterozygosity’ and ‘length effect’ used in our linear model of recombination. (0.09 MB DOC) [file pone.0008650.s001.doc]

## Indel recombination model

In this section, we derive the effects of an indel on the recombination rate. We also give details about the co-variates we used in the linear models.

*Background*

LDhat estimates $\rho=4N_{e}r=4R/L$ where *L* is the length of a window (here *L*=386 bp) and *r* is the recombination rate per nucleotide per generation. (LDhat is using the distance between SNPs as *L* but we subsequently rescale to have equal lengths of all windows (see Material and Methods)). Importantly, in the Seattle SNP and NIEHS data sets the distance between two sites with a polymorphic indel between them is defined using the long variant of the indel as reference. For instance, if two SNPs are adjacent in some haplotypes but separated by 2 bp in the other haplotypes, then the distance between the SNPs is 0 in some haplotypes but 2 in other haplotypes. However, if the first SNP has position 1000, the indel will occupy positions 1001 and 1002 and the second SNP will be assigned position 1003 regardless of the frequency and ancestry of the indel. This affects the estimated recombination rate and we denote this by the *length effect*. Additionally, the recombination rate may be suppressed in individuals heterozygote for the indel – the *heterozygosity effect*. To model these effects jointly, consider a window containing a polymorphic indel and denote the frequency of the long indel variant by *p* and its length by *l*. Denote the decrease, in heterozygotes relative to homozygotes, of the recombination rate per site in a window by *c*. Then the expected number of recombination events in one generation is

- in homozygotes for the long indel variant: $4N_{e}rL$

- in homozygotes for the short indel variant: $4N_{e}r(L-l)$

- in heterozygotes: $4N_{e}r(L-0.5l)(1-c)$

[Note that this parameterization implies that $c\leq1$ and that it allows, when c is negative, for an increase in recombination rate in heterozygotes. Here we will assume that $\left|c\right|<1$although $c<-1$would be theoretically possible (implying a more than 2-fold increase in recombination rate in heterozygotes).] At the population level, computing a weighted average of these three categories using the Hardy-Weinberg frequencies by effectively summing up the three terms above yields

$4N_{e}r(L-l(1-p)-c2p(1-p)(L-0.5l))$.

This is $4N_{e}r(l(1-p)+c2p(1-p)(L-0.5l))$fewer events than the $4R=4N_{e}rL$expected in windows without indels.

*Effect in linear model*

In order to incorporate these effect in our linear model, we are searching for a function *f(l,p,c)* of *l*, *p* and *c* that will capture this change such that

$\ln\left(\frac{4R}{L}\right)+f(l,p,c)=\ln\left(\frac{4N_{e}r(L-l(1-p)-c2p(1-p)(L-0.5l))}{L}\right)$.

Solving for *f(l,p,c)*, we get

$f(l,p,c)=\ln\left(1-\frac{l}{L}(1-p)-c2p(1-p)\frac{2L-l}{2L}\right)$.

Furthermore, using the Cauchy-Schwarz inequality

$\left|\frac{l}{L}(1-p)+c2p(1-p)\frac{2L-l}{2L}\right|^{2}\leq\left(\left|\frac{l}{L}(1-p)\right|+\left|c2p(1-p)\frac{2L-l}{2L}\right|\right)^{2}<\left(\frac{l}{L}+\left|c\right|0.5\frac{2L-l}{2L}\right)^{2}<\left(\frac{2L+3l}{4L}\right)^{2}$which is smaller than 1 if $l\leq2L/3$. This is certainly true so we can write

$$f(l,p,c)=-\sum_{n=1}^{\infty} \frac{1}{n}\left(\frac{l}{L}(1-p)+c2p(1-p)\frac{2L-l}{2L}\right)^{n}$$

Ignoring higher order terms we approximate this by

$f(l,c,p)\approx-\frac{l}{L}(1-p)-c2p(1-p)$.

This approximation can be discussed but should be acceptable since *l* is much smaller than *L* and *p* is usually close to 0 or close to 1. Moreover, it conceptually captures the two effects we are after: in windows with polymorphic indels the recombination rate is expected to decrease by approximately $l(1-p)/L$ due to the fact that there are fewer sites to recombine in - the length effect, and to decrease by $c2p(1-p)$ if the actual recombination rate per site is lower in heterozygotes, the heterozygosity effect. Since *L* and 2*c* are constants, we use $l(1-p)$and $p(1-p)$to test separately for the length- and heterozygosity effect in our linear models.

To be more precise concerning the length effect, assume that there is no suppression of recombination in heterozygotes, the average population length of a window is not *L* but$pL+(1-p)(L-l)=L-l(1-p)$and the number of excepted recombination events per generation is$4N_{e}r(L-l(1-p))$. If we want to estimate the scaled recombination rate $4N_{e}r$we should divide the number of recombination events by$L-l(1-p)$. When dividing by *L* instead, we underestimate $4N_{e}r$since$L>L-l(1-p)$. The underestimate in our log-transformed estimate of the recombination rate is thus

$$\ln\left(\frac{4R}{L-(1-p)l}\right)-\ln\left(\frac{4R}{L}\right)=-\ln\left(1-\frac{l}{L}(1-p)\right)=\sum_{n=1}^{\infty} \frac{1}{n}\left(\frac{l(1-p)}{L}\right)^{n}\approx\frac{l}{L}(1-p)$$

In this sense, the length effect is better perceived as an artifact and is necessarily present. To get an upper bound of the effect, notice that we overestimate the recombination rate if instead of defining the distance between sites relative to the long indel variant we define it relative to the short indel variant. So the maximum error we make is smaller than$\left|\ln\left(\frac{4N_{e}r(L-l)}{L}\right)-\ln\left(\frac{4N_{e}rL}{L-l}\right)\right|=-2\ln\left(1-\frac{l}{L}\right)$. In our data L=386 and the average length of indels is smaller than 3.5 bp (3.17 for insertions and 3.46 for deletions, data not shown) so the maximum error is smaller than $-2\ln\left(1-3.5/386\right)\approx0.018$. The variance and mean of the log transformed recombination rate in windows without polymorphic indels is 3.488 and -1.546 respectively (data not shown) so the maximum length effect is around 1.2 % of the mean and 0.5 % of the variance.

Note that this approach only describes the effect of an indel on recombination rate at a specific time. The correct thing to do would be to integrate over the sample history with respect to the heterozygosity and the frequency of the long indel variant but besides being difficult, most of the recombination events that affect the sample are expected to happen when there are many branches in the ancestral recombination graph, *i.e.* recently in the sample history. Thus using the current sample frequency of indels is a sensible approximation. Notice however that when the indel is a deletion, *p* is the frequency of the ancestral variant but the derived frequency if the indel is an insertion. Since the derived frequency is typically low and decreasing back in time, this means that *i)*$1-p$is on average close to zero and decreasing for deletions but, *ii)* $1-p$is close to one and increasing for insertions. This means that the length effect should be easier to detect for insertions than for deletions and that it is underestimated for insertions but overestimated for deletions. Finally, we are overestimating the heterozygosity effect as the average trajectory of *p* drifts away from 0.5 since the average *p* for deletions is large and increasing but small and decreasing for insertions.

Taking more than one indel polymorphism into account is more difficult as one has to consider how they are linked and in these cases we used the largest value of $p(1-p)$ and the largest value of $l(1-p)$ over the indels in the window.
