# Supplementary material for: Insertion and Deletion Processes in Recent Human History
Source: PLoS One. 2010 Jan 19;5(1):e8650. doi: 10.1371/journal.pone.0008650 (PMC2808225; doi:10.1371/journal.pone.0008650)

## Data filtering and possible biases

Eight genes were sequenced in both the NIEHS and Seattle SNPs project. These were IGF1, LTA, LTB, MMP9, PTGS2, TGFB3, TNF and TNFRSF1B. For these we used the version retrieved from NIEHS in all cases except IGF1 and MMP9. The gene LPA retrieved from Seattle SNPs was considered badly aligned as the provided positions in ENCODE did not seem to make sense and was discarded from the analysis. Moreover, mapping positions in NIEHS and Seattle SNPs to positions in the reference sequences in UCSC was problematic for some genes and these were removed (26 genes: removed due to problems in mapping positions in Seattle SNP to positions in the reference sequences in UCSC: F2RL1, F9, GP1BA, IGF1, IL3RA, IL4, KNG, PPARA, SMP1; due to problems in mapping positions in NIEHS to positions in the reference sequences in UCSC: BCL2, CDK7, CHC1L, COMT, ERCC3, FLJ35220, GCSH, MDM2, MSH3, NQO1, PMS1, POLB, POLG, RFC5, TDG, XDH, XPC.)

The major effort in the parsing process was to avoid producing systematic biases. We do not claim that we have entirely gotten rid of all biases as some biases may in fact be unavoidable. As a hypothetical example, if insertions are more often associated with regions of low complexity than deletions, we have most certainly selected for deletions in our parsing as alignments of low complexity regions are generally of lower quality. To avoid this class of artifacts, we have explicitly geared our analysis towards comparing categories instead of estimating parameters.

**The influence of microsatellites**

Another issue is the magnitude of the influence of microsatellites on our data. We have not explicitily avoided this class of variation but our filtering procedure

will a priori exclude long (hyper mutable) microsatellites. The possibility that more indels make up a greater proportion in the fixed human category than in the polymorphic category - and in this way biasing the DI-ratio comparison – is unlikely. This is because the regions for which we are scanning for fixed human and chimp indels are precisely the regions that were resequenced in the Seattle SNPs and NIEHS programs. However, to more specifically test this, we defined as ‘microsatellite-like’ regions we used in our study that contained motifs repeated at least four times and which constitute at least 10 bp (this corresponds to less than 1% of the investigated genomic regions). Contrasting indels overlapping with regions annotated as ‘microsatellite-like’ with those that do not and polymorphic with fixed human indels in a 2x2 contingency table we find no significant difference either for insertions or deletions (Table S2). Insertions were however markedly overrepresented in microsatellite regions (about 11 % of insertions overlap with microsatellites while only 4 % of deletions do so).

**More stringent indel filtering**

The argument that we parsed correctly relies on extensive manual checking and on the fact that increasing the stringency, either by requiring fewer differences in the flanking regions or by removing 1 bp indels and poly (A/T) indels, did not qualitatively change the results:

*Alternative indel filtering conditions 1 (“Cond1”)*: Table S3 and Figure S1. Indels longer than 1 bp and indel motif not only A’s or only T’s. Same stringency of flanking regions as in main article: for polymorphic indels, we required that 28 out of 32 flanking positions were identical in the human-chimpanzee alignment and also that there were no gaps at these positions. We also required that either all human sites were gaps or that all chimp sites were gaps within the indel sites in the human-chimpanzee alignment. For fixed indels, we added the additional requirements that 20 out of 32 directly adjacent flanking positions were identical in the human-chimpanzee-macaque alignment and that there were no gaps at these positions. No ambiguous information was allowed within the indel sites in the human-chimpanzee-macaque alignment.

*Alternative indel filtering conditions 2 (“Cond2”)*: Table S4 and Figure S2.Stringency of flanking regions higher than in main article: for polymorphic indels, we required that 30 out of 32 flanking positions were identical in the human-chimpanzee alignment and also that there were no gaps at these positions. We also required that either all human sites were gaps or that all chimp sites were gaps within the indel sites in the human-chimpanzee alignment. For fixed indels, we added the additional requirements that 28 out of 32 directly adjacent flanking positions were identical in the human-chimpanzee-macaque alignment and that there were no gaps in 64 directly adjacent sites (twice the length compared to Cond1 and main article). No ambiguous information was allowed within the indel sites in the human-chimpanzee-macaque alignment.

**Table S2**. Adapted McDonald-Kreitman test of difference between indels within and outside ‘microsatellite-like’ regions for the fixed human and polymorphic human data categories.

|  | Deletions | | Insertions | |
| --- | --- | --- | --- | --- |
|  | inside ”microsats” | Outside ”microsats” | inside ”microsats” | outside ”microsats” |
| Fixed Human | 139 | 2860 | 282 | 2346 |
| Polym Human | 171 | 4211 | 207 | 1639 |
| $\chi^{2}$ | 2.37 (not significant) | | 0.26 (not significant) | |

**Table S3**. After filtering according to Cond1. Adapted McDonald-Kreitman test of differences between insertions and deletions in fixed human and polymorphic human data categories.

|  | Deletions | Insertions | Ratio |
| --- | --- | --- | --- |
| Fixed Human | 1518 | 652 | 2.33 |
| Polym Human | 2367 | 679 | 3.49 |
| $\chi^{2}$ | 40.09 ^***^ | | |

**Table S4**. After filtering according to Cond2. Adapted McDonald-Kreitman test of differences between insertions and deletions in fixed human and polymorphic human data categories.

|  | Deletions | Insertions | Ratio |
| --- | --- | --- | --- |
| Fixed Human | 1933 | 1119 | 1.73 |
| Polym Human | 4331 | 1816 | 2.38 |
| $\chi^{2}$ | 47.61 ^***^ | | |

**Figure S1**. After filtering according to Cond1. Deletion count (top), insertion count (middle) and DI-ratio (bottom) for different indel lengths (in bp) (white: polymorphic human, grey: fixed human, black: chimp). Note the difference in scale on the y-axis for indels of length 1 to 4 bp and those longer in the two top graphs.


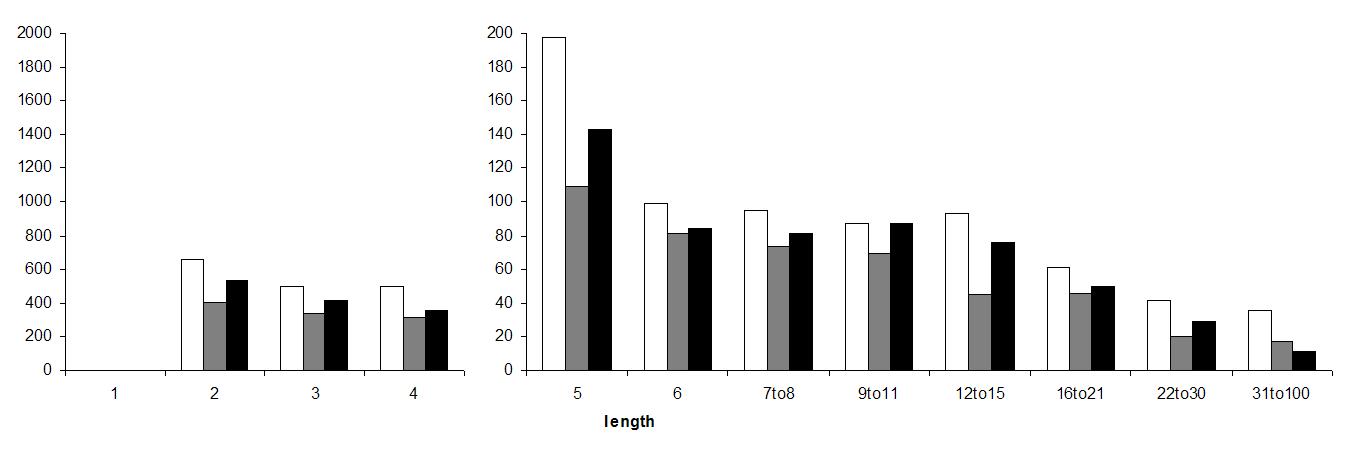

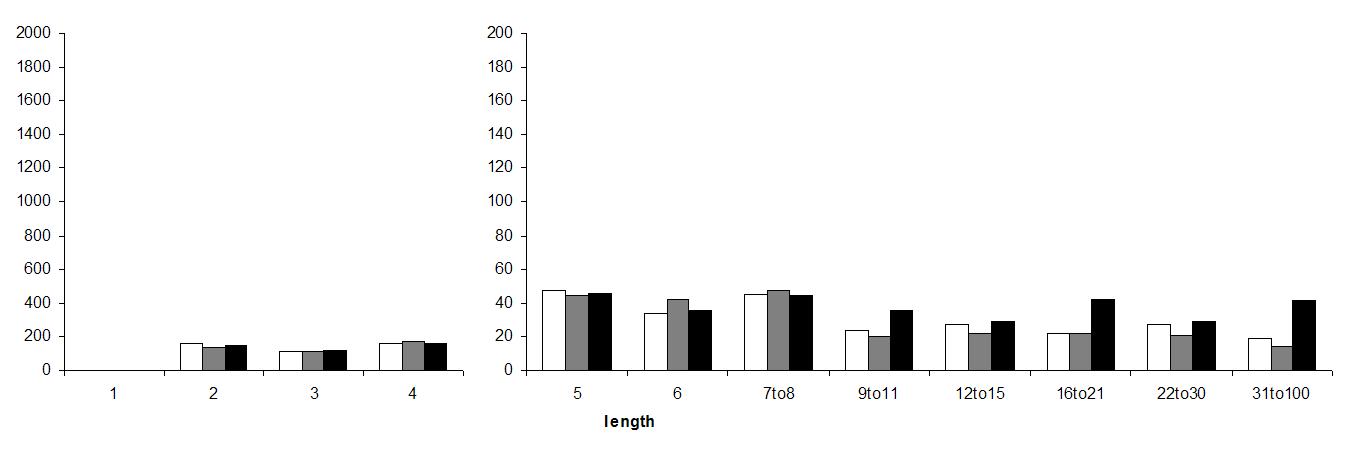


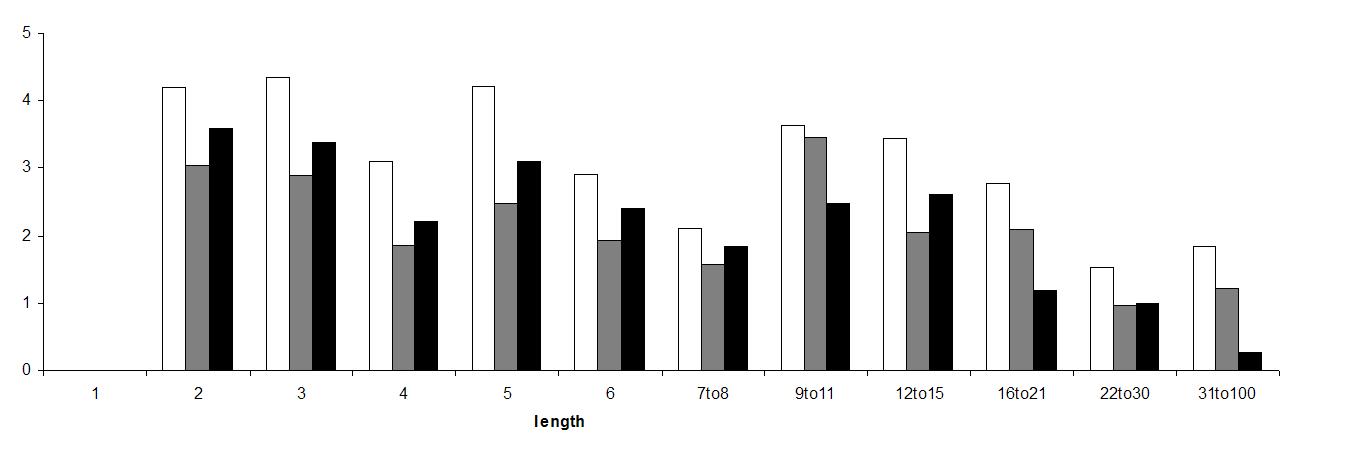


**Figure S2**. After filtering according to Cond2. Deletion count (top), insertion count (middle) and DI-ratio (bottom) for different indel lengths (in bp) (white: polymorphic human, grey: fixed human, black: chimp). Note the difference in scale on the y-axis for indels of length 1 to 4 bp and those longer in the two top graphs.


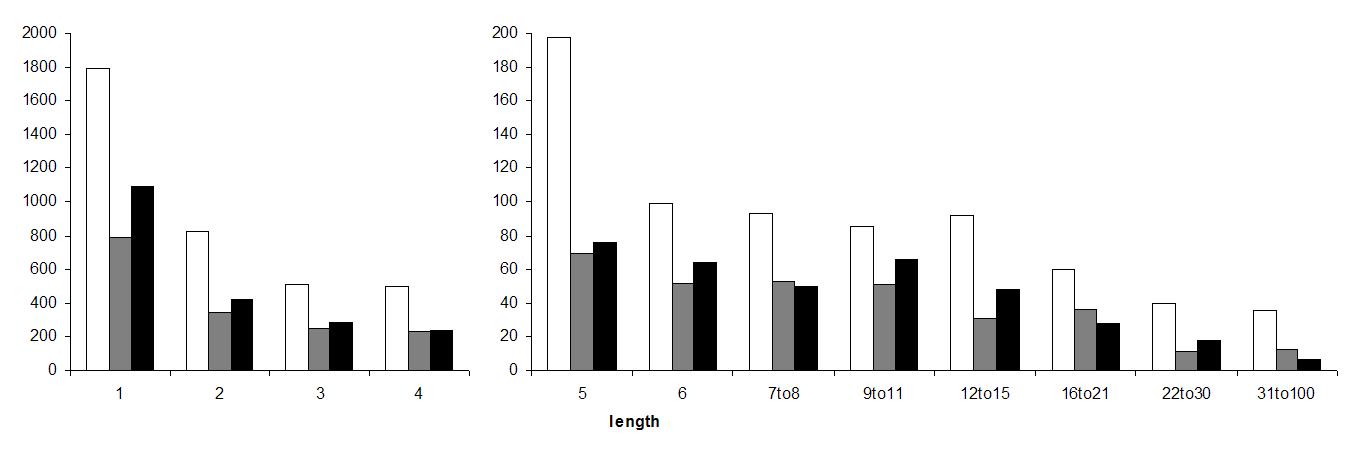

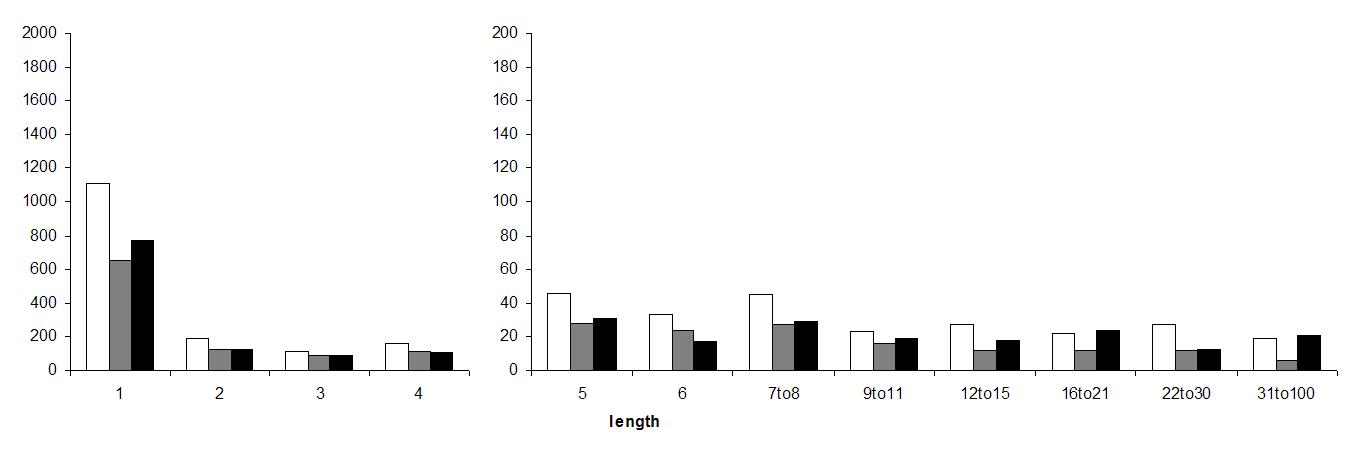

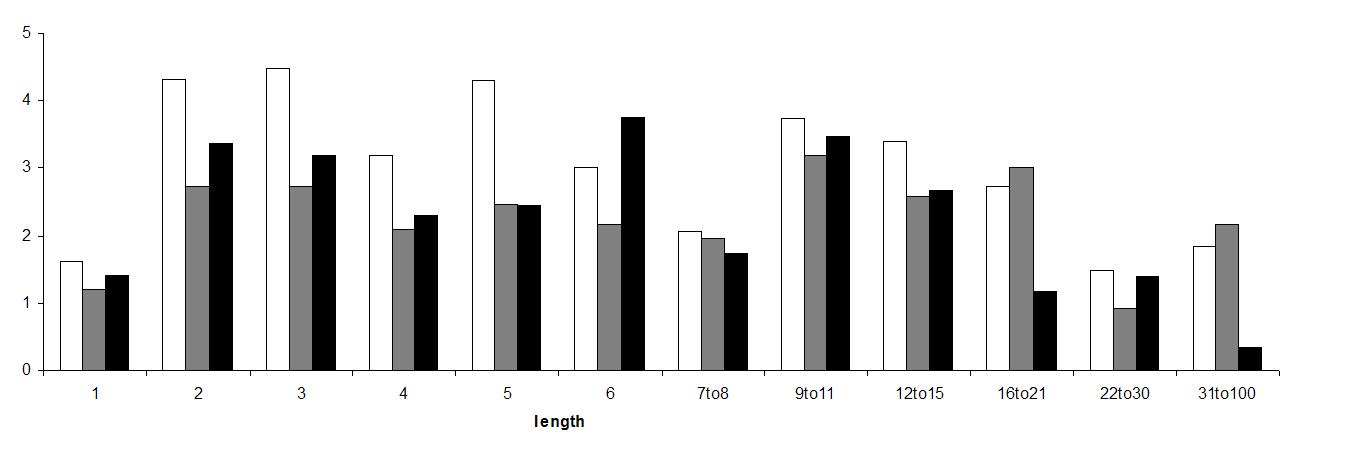

Supplement: Text S2 — Data filtering and possible biases. Discussion of possible non-biological sources of our results. (0.27 MB DOC) [file pone.0008650.s002.doc]
