# Supplementary material for: Insertion and Deletion Processes in Recent Human History
Source: PLoS One. 2010 Jan 19;5(1):e8650. doi: 10.1371/journal.pone.0008650 (PMC2808225; doi:10.1371/journal.pone.0008650)
Supplement: Table S1 — Trained recombination model. Table showing specifications of the trained recombination model. (0.08 MB DOC) [file pone.0008650.s003.doc]

**Table S1**. Summary of the variable affecting local recombination rates

All windows containing more than 90% scanned sites and without polymorphic indels were used (42214 windows). We report the linear 2-way interaction model of the log-tranformed recombination rate after a ‘step’ call. Model’s adjusted R^2^ =0.1264.

|  | Effect | p-value |
| --- | --- | --- |
| GC | + | < 10^-15^ |
| AT→GC count | + | < 10^-15^ |
| UTR | + | < 10^-15^ |
| CDS | - | 3.2 x 10^-14^ |
| Chomosome X | - | 1.6 x 10^-12^ |
| polyAT : SINE | - | 1.3 x 10^-8^ |
| CpG : SINE | - | 7.0 x 10^-8^ |
| GC : UTR | - | 8.7 x 10^-8^ |
| polyAT | + | 2.1 x 10^-6^ |
| GC : CpG | - | 8.6 x 10^-6^ |
| SINE : telomere distance | - | 8.8 x 10^-6^ |
| SINE | + | 8.9 x 10^-6^ |
| SINE : Repeat masked^b^ | + | 1.4 x 10^-5^ |
| GC : CDS | + | 1.5 x 10^-5^ |
| GC : polyAT | - | 2.3 x 10^-5^ |
| UTR : Chromosome X | - | 2.9 x 10^-5^ |
| Other^a^ count | + | 3.2 x 10^-5^ |
| Telomere distance | - | 2.3 x 10^-4^ |
| GC : Repeat masked^b^ | - | 2.4 x 10^-4^ |
| CDS : Not scanned | - | 3.1 x 10^-4^ |
| LINE | - | 4.2 x 10^-4^ |
| Chromosome X : telomere distance | + | 0.0012 |
| Other^a^ count : GC | - | 0.0016 |
| Repeat masked^b^ | + | 0.0021 |
| CDS : UTR | + | 0.0026 |
| CpG | + | 0.0037 |
| CDS : telomere distance | + | 0.0042 |
| CpG : telomere distance | - | 0.0060 |
| GC : LINE | + | 0.0066 |
| polyAT : CpG | + | 0.0084 |
| UTR : SINE | - | 0.0087 |
| Other^a^ count : polyAT | - | 0.011 |
| CpG : CDS | + | 0.015 |
| Other^a^ count : Chromosome X | + | 0.016 |
| Repeat masked^b^ : telomere distance | - | 0.017 |
| Repeat masked^b^ : Not scanned | - | 0.024 |
| CDS : Chromosome X | + | 0.028 |
| AT→GC count : telomere distance | - | 0.030 |
| CpG : Chromosome X | + | 0.045 |
| AT→GC count : LINE | + | 0.047 |
| polyAT : Chromosome X | - | 0.055 |
| Not scanned | - | 0.089 |
| SINE : LINE | + | 0.099 |
| SINE : Not scanned | + | 0.11 |
| CpG : Repeat masked^b^ | + | 0.11 |
| GC : telomere distance | + | 0.14 |
| LINE : Chromosome X | + | 0.14 |
| polyAT : LINE | + | 0.15 |

^a^: single nucleotide changes not (A or T) → (G or C), ^b^: not due to SINEs or LINEs
